# Harmonizing gene nomenclature and disease classification for ADSS1 myopathy

**DOI:** 10.1186/s13023-026-04535-8

**Published:** 2026-08-05

**Authors:** Emma Rybalka, Oleg A. Shchelochkov, Merve Koç Yekedüz, Jaymin Upadhyay, Alan H. Beggs

**Affiliations:** 1 Inherited and Acquired Myopathies Program, Institute of Health and Sport, Victoria University, Melbourne, VIC Australia; 2 Melbourne Medical School – Western Health, Department of Medicine, University of Melbourne, Melbourne, VIC Australia; 3 Cure ADSSL1, Glendale, CA USA; 4 Purine and Pyrimidine Metabolism Unit, National Human Genome Research Institute, National Institutes of Health, Bethesda, MD USA; 5 Department of Anesthesiology, Critical Care and Pain Medicine, Boston Children’s Hospital, Harvard Medical School, Boston, MA USA; 6 Faculty of Medicine, Department of Pediatric Metabolism, Ankara University, Ankara, Türkiye; 7 Ankara University Rare Diseases Application and Research Center, Ankara, Türkiye; 8 Division of Genetics and Genomics, The Manton Center for Orphan Disease Research, Boston Children’s Hospital, Harvard Medical School, Boston, MA USA

**Keywords:** Adenylosuccinate synthase 1 deficient myopathy, ADSS1, ADSSL1, PURA1, Metabolic myopathy, Purine metabolism

## Abstract

ADSS1 myopathy is an ultra-rare autosomal recessive neuromuscular disorder caused by biallelic pathogenic variants in *ADSS1* encoding the striated muscle isozyme of adenylosuccinate synthase involved in purine metabolism. Despite advances in clinical, genetic and mechanistic understanding, the condition remains inconsistently represented across clinical, genomic and registry systems. It is variably labelled as ADSSL1-related distal myopathy, nemaline myopathy, or more recently, a metabolic myopathy reflecting an inborn error of metabolism. These inconsistencies have practical consequences. Gene nomenclature and annotation drift introduces avoidable errors in variant interpretation, panel design and data integration, while imprecise disease classification based on early observations reduces clinical ascertainment of new cases, degrades longitudinal natural history studies, and delays clinical trial readiness. At the level of international coding systems, ADSS1 myopathy is misclassified under legacy terminology in SNOMED CT and Orphanet and is not independently represented in ICD-11. This position statement argues for harmonization of both gene and variant nomenclature and disease classification. We promote adoption of *ADSS1* (HGNC:20093) as the canonical gene symbol, adenylosuccinate synthase 1 (ADSS1) as the canonical protein name, with ADSSL1 retained as a historical synonym, and PURA1 recognized as a protein alias. We further propose classification of ADSS1 myopathy as a metabolic myopathy caused by an inborn error of purine metabolism (more concisely, a metabolic myopathy), with distal and nemaline descriptors retained as histopathological modifiers. Standardization is a low-cost, high-impact intervention that will improve diagnostic accuracy, enable integration of multi-omics datasets, strengthen registries and accelerate therapeutic development for this globally dispersed ultra-rare disease.

**Clinical trial number:** Not applicable.

## Introduction

Rare diseases are characterized by scarcity of patients, data, and clinical familiarity. In this setting, consistent gene nomenclature and disease classification underpin diagnosis, data aggregation and translational progress. When terminology is inconsistent, patients may be overlooked, datasets fragmented, and clinical reasoning shaped by incomplete or outdated frameworks. These challenges are magnified in ultra-rare conditions, where each individual case contributes substantially to defining disease biology and longitudinal natural history.

Adenylosuccinate synthase 1 deficiency — hereafter referred to as ADSS1 myopathy – exemplifies this problem. This ultra-rare neuromuscular disorder characterized by progressive muscle weakness, loss of function and early mortality, has high unmet need. As outlined in our recent global meeting report, the condition is under-recognized despite increasing international case identification [1]. This under-recognition may be driven in part by inconsistencies in gene naming, disease classification and database representation.

## Gene and protein nomenclature

The gene encoding the muscle enriched isozyme of adenylosuccinate synthase is formally designated *ADSS1* (HGNC:20093) and located on chromosome 14q32.33. It is distinct from – and expressed > 100-fold more abundantly than – the ubiquitously expressed *ADSS2* (HGNC:292; chromosome 1q44) [2]. The distinction is biologically meaningful, reflecting tissue-specific regulation of purine metabolism. The legacy symbol *ADSSL1* originated during early genome annotation based on sequence homology [3, 4] and persists across databases and literature. While historically valid, its continued use fragments variant annotation and literature retrieval.

The existence of multiple transcripts for many genes can lead to additional confusion when diagnostic findings are reported relative to different isoforms. The Matched Annotation from NCBI and EMBL-EBI (MANE) transcript set was developed to identify and annotate clinically relevant transcripts and ensure consistency in variant reporting [5]. In the case of *ADSS1*, the first instances of myopathy-related variants were published prior to the release of the MANE transcript set and annotated relative to RefSeq transcript NM_199165 [6]. However, later reports recognized that this transcript is not expressed at detectable levels in striated muscles and utilized the now-current MANE transcript, NM_152328 [7–10]. Unfortunately, confusion remains, with publications continuing to report variants based on NM_199165, and in some cases even reporting multiple patients with the same variant as having “different” pathogenic variants based on undocumented use of both transcripts [11, 12]. To achieve clarity, it is essential that every report of *ADSS1* genotypes specify the reference transcript being utilized, and whenever possible should number variants relative to the then current muscle-expressed MANE transcript (NM_152328.5) and its associated protein (NP_689541.1).

A third layer of confusion arises from protein-level annotation. In UniProt, the *ADSS1* gene is associated with the entry name PURA1_HUMAN, often abbreviated to “PURA1” in proteomic datasets (Q8N142). This is not a gene symbol but a mnemonic identifier. In practice, however, it is frequently interpreted as such, leading to a disconnection between genomic and proteomic datasets. The potential for confusion is compounded by similarity to *PURA*, which encodes the transcription factor Pur-α. To harmonize protein nomenclature with the corresponding gene symbol in accordance with current international naming guidelines, we propose adopting “ADSS1” as the universal abbreviation for adenylosuccinate synthase isozyme 1 from any species (https://www.ncbi.nlm.nih.gov/genbank/internatprot_nomenguide/). We recognize, however, that the historically used form “AdSS1” has appeared in both earlier [4] and recent [13] publications. As this capitalization does not affect database retrieval, we do not discourage its continued use but recommend ADSS1 to promote consistency across genomic, proteomic and biomedical databases. As multi-omics approaches become increasingly central to rare disease research, consistent identifier mapping is essential. Failure to reconcile ADSS1, ADSSL1 and PURA1 across platforms risks obscuring biological signals and therapeutic targets.

## Disease classification

ADSS1 myopathy was initially described in 2016 as an adolescent-onset distal myopathy [6] and classified as *myopathy*,* distal*,* 5* (MPD5) [6]. Subsequent studies have demonstrated a broader phenotype, including proximal, axial, bulbar and cardiorespiratory involvement, with heterogeneous progression [7, 12, 14, 15]. In one large series, well over half of patients were reported to have proximal weakness equal to or greater than distal involvement [10]. A purely distal classification is therefore misleading, risks misclassification and introduces a clinical ascertainment bias.

The classification of ADSS1 myopathy as a nemaline myopathy is similarly problematic. Although nemaline rods have been observed in a subset of biopsies [7, 10, 16], they are neither universal nor specific. Furthermore, nemaline myopathy is associated with at least 11 genes, all which regulate sarcomere thin filament structure and dynamics rather than metabolic enzymes [17, 18]. In the context of ADSS1 myopathy, nemaline bodies are best interpreted as secondary histopathological modifiers related to muscle stress rather than pathology defining.

By contrast, classification as a metabolic myopathy is consistent with both mechanism and phenotype [19] which would establish this “purine metabolic myopathy” as a fourth subcategory alongside the three classical subsets, namely glycogen storage disorders, fatty acid oxidation defects, and mitochondrial disorders. ADSS1 catalyzes a key step in de novo purine nucleotide metabolism synthesizing adenylosuccinate from inosine monophosphate [4], and loss of function disrupts AMP synthesis and energy homeostasis in animal models [13, 20] (yet to be validated in human muscle). This disruption represents a classical inborn error of metabolism, and as such, ADSS1 myopathy is captured among disorders of purine metabolism in standard medical references [21]. Since ADSS1 is highly enriched in muscle, the clinical features are distinctly myopathic: muscle weakness, exercise intolerance, elevated (mild-moderate) creatine kinase levels, myopathic electromyographic findings and structural myopathic changes on muscle biopsy including lipid deposition, vacuolar and mitochondrial abnormalities are reported [7, 9, 12]. Indeed, recent clinical imaging studies reveal widespread muscle lipidosis as the most homogenous feature [8, 14, 22]. Thus, there is considerable clinical overlap with classical metabolic myopathy subtypes. Although not a classification-defining aspect per se, rational therapeutic strategies are also aligned with classical metabolic myopathies [23–25]. A Phase 1b trial of adenylosuccinic acid (ASA; NCT07412821) is based on metabolic bypass of enzyme deficiency, but enzyme replacement through infusion or gene modification is also logical.

Since ADSS1 myopathy occupies a unique position at the interface between inherited metabolic disorder and muscle disease, we propose reclassification as a metabolic myopathy to capture both the biochemical basis and the predominant clinical manifestations of this disorder.

## System-level misclassification

Nomenclature inconsistencies are compounded at the level of clinical coding and disease ontology systems, which underpin diagnosis, registry development, epidemiology, and health service planning. ADSS1 myopathy is currently represented in SNOMED CT (SCTID: 1172694007) and Orphanet (ORPHA: 482601) as ADSSL1-related distal myopathy, reflecting both early phenotype-based descriptions and the continued use of historical gene nomenclature rather than current biological understanding. SNOMED CT underpins diagnostic coding within electronic health records, while Orphanet provides the primary reference framework for rare disease classification and registry alignment. In contrast, ICD-11, maintained by the World Health Organization, serves as the global standard for disease classification in epidemiology, and health system reporting. ADSS1 myopathy is not independently represented in ICD-10/11 and is instead subsumed within broader neuromuscular categories (G71.0: Muscular Dystrophy).

Accurate classification within these systems is critical. Coding frameworks determine how diseases are recognized, counted, and linked across clinical, research, and policy environments. Misclassification as a distal or nemaline myopathy risks inappropriate diagnostic pathways, clinical under-recognition, underestimation of disease burden, and exclusion from relevant registries and clinical trials. At a systems level, inconsistent classification limits interoperability and reduces the visibility of the condition in health policy and resource allocation. An outcome of this position statement will be to harmonize ADSS1 myopathy entries across these databases.

## Global implications for ultra-rare disease

ADSS1 myopathy is globally distributed, with patients identified across multiple countries, healthcare systems and languages [1]. In this context, inconsistent nomenclature prevents recognition that cases are related, limiting data aggregation and delaying natural history studies. Clinical trial readiness is also impacted. Recruitment for ultra-rare disease trials depends on identifying small numbers of eligible individuals across international registries. Variability in naming can lead to under-ascertainment and exclusion of patients who would otherwise be eligible. These challenges align with broader international priorities, including initiatives led by the National Institutes of Health and the European Joint Programme on Rare Diseases, which emphasize standardized terminology as a foundation for cross-border data sharing and translational research [26, 27].

## Recommendations

We propose the following harmonized framework:

1. **Disease name**: ADSS1-deficient myopathy, abbreviated as ADSS1 myopathy.
2. **Gene nomenclature**: *ADSS1* (HGNC:20093) as the canonical symbol.
3. **Clinical genetic variant reporting**: utilizing the MANE transcript NM_152328.5, corresponding to ENST00000330877.7, and its corresponding reference protein NP_689541.1.
4. **Protein nomenclature**: adenylosuccinate synthase 1 (ADSS1) as the canonical protein name.
5. **Synonyms**: ADSSL1 retained as historical; PURA1 recognized only as a protein alias, otherwise its use completely abandoned.
6. **Disease classification**: Metabolic myopathy caused by an inborn error of purine metabolism, abbreviated as metabolic myopathy.
7. **Descriptors**: the use of distal myopathy as a descriptor for the overall condition should be retired; the terms “nemaline”, “rimmed-vacuolar” and “lipid/mitochondrial changes” acknowledged as non-defining histopathological modifiers.
8. **A stylistic example given on usage is**: “We sequenced *ADSS1* (NM_152328.5, corresponding to ENST00000330877.7) and assessed ADSS1 (NP_689541.1) abundance by immunoblot in a cohort diagnosed with ADSS1 myopathy, a metabolic myopathy caused by an inborn error of purine metabolism.” Implementation of this framework across journals, databases, clinical laboratories and registries is both feasible and urgent.

## Conclusion

ADSS1 myopathy illustrates how historical nomenclature and evolving phenotype can create a fragmented and clinically confusing landscape. This disease also highlights a broader challenge in the classification of genetic myopathies that do not neatly fit within any sub-type paradigm. Since the underlying biology and clinical spectrum of ADSS1 myopathy is now much clearer, aligning gene and disease naming with this understanding is a simple but impactful step. Standardization will improve diagnostic accuracy, enable integration of multi-omics data, strengthen global registries and accelerate therapeutic development. Most importantly, it will ensure that patients with this ultra-rare condition are recognized, connected and appropriately managed. In ultra-rare disease, getting the name right is an essential part of getting the diagnosis – and ultimately the treatment – right. 

## Data Availability

No datasets were generated or analysed during the current study.
